# Consent in Surgical Operations

**Published:** 1896-02

**Authors:** 


### CONSENT IN SURGICAL OPERATIONS.

The surgeon is placed in a situation of great delicacy and embarrassment when the family of his patient, or some member of it, offers objection to the performance of an operation, the necessity for which is clearly indicated and recognized by the physician. Especially distressing is it when the patient is a wife and mother, on whose life and health depends the home, and, as is most common and most unfortunate, the objection is raised by the husband. If demanded to save the life and the patient is conscious and sane and consents, the operation should be done no matter from what source the objections or who may be the objectors. The husband, by virtue of his position as head of the family, should have the privilege to choose the surgeon. It must not be concluded that he has no rights which are bound to be respected; that the solicitude natural to his relationship should not be honored. It is only where he is governed by selfish motives; where he values his wife but little more than he does his horse; where unreasoning prejudice or extraneous evil influence prompts his opposition, that he should be pushed aside and the welfare and will of the wife alone considered.

Few surgeons are willing to undertake a case where the husband or family prefer that the patient die, or pass into chronic invalidism and lifelong suffering, rather than submit to an operation which would save life, increase the chances for ultimate recovery, or even alleviate suffering. For where he encounters such objections, the surgeon is not only subjected to increased anxiety in his surgical work, but he incurs the risk of possible annoyance, perhaps to extent of pecuniary mulcting. There is too much needy and exacting work ready at hand, responsibility is too onerous, life is too short, to wait and worry and risk in cases where confidence is wanting and capability is unrecognized; where the work and its incident responsibilities are unappreciated, and difficulties and embarrassments are thrown in the way of its successful execution.

Aside from the feelings of the family are other factors which operate to influence the patient: the talk of the neighbors, lineal descendants of the comforters of Job; the gossip, who can give details of the operation and foretell the result—some one she knew told her all about a case somebody had heard some one else relate; the corner druggist, who kindly volunteers an opinion based upon his professional knowledge. Patients have been deceived as to real conditions by men anxious to operate or greedy for large fees. This fact often influences a patient and family, especially where the outward manifestations of disease are not indicative of its gravity. There is an almost irresistible tendency to wait, in hope that "something will turn up" for the better. The profession in general recognizes that in many surgical cases, delay proves disastrous; that if it is not fatal, it results in chronic invalidism and places the patient beyond the possibility of complete recovery. In some cases more or less responsibility attaches to the attending physician who, ignorant of the real trouble or influenced by some questionable motive, discourages operation and even examination by some specialist in the suspected condition.

On the other hand, the surgeon must feel confident of his position. There are errors committed so wilfully, selfishly and wantonly, that even that charity which "covereth a multitude of sins" can not palliate them. Surgery, and particularly gynæcological surgery, is full of temptations seductive to one ambitious for name and gain; temptations which appeal to many very weak points in human nature. The true surgeon will obliterate thoughts of self and consider the issues of his work. These are momentous, for they deal with human life, with time and with eternity. No man can envy success gained in the way indicated by the dying declaration of a distinguished specialist, operator, writer and teacher, that more than one-half of his operations were done for money. The lesson in this confession should sink deep into the surgical mind, and yet deeper into the surgical conscience. When there is less of needless meddlesome operative interference, less of ignorant blundering work done, there will be less of objection raised where conditions indicate the necessity of resorting to surgery.

Legally considered, to justify a surgical operation upon a married woman, her consent and not that of her husband is necessary. A married woman cannot be compelled to submit to an operation; but if she voluntarily submits to its performance her consent will be presumed, unless she was the victim of fraudulent misrepresentation. Even if the disease resulting in death was caused by the operation, the surgeon is not liable if he performed the operation with the patient's consent, in a careful and skillful manner, and under the belief that it was proper to be performed.

The party who allows a surgical operation to be performed is presumed to have employed the surgeon for that particular purpose, and it will be presumed that the operation was carefully and skillfully performed in the absence of proof to the contrary.

A judge of the supreme court of Maryland, rendering a decision in a case in point, tersely and aptly states the principle governing in the matter of consent:

"Surely the law does not authorize the husband to say to his wife 'You shall die of cancer; you cannot be cured, and a surgical operation affording only temporary relief, will result in useless expense.' The husband has no power to withhold from his wife the medical assistance which her case might require. The consent of the wife, not that of that husband was necessary."

It was said by the supreme court of Michigan, in a recent case:

"It would be a cruel rule for her, the wife, if she cannot, in the absence of the husband at least, or in his presence if he does not himself provide for her, make a binding agreement for any necessaries, whether articles to be provided, or professional help. It is not to be expected that physicians and surgeons will always feel bound to render gratuitous treatment to injured persons, and when the occasion is pressing, it would be unreasonable to delay until an absent husband is communicated with to learn whether he consents or refuses to assume her contracts. Time will not allow minute inquiries, humanity will not prompt them. It seems to us that no sensible line can be drawn between contracts for food and clothing and contracts for medical aid.—*Medical and Surgical Reporter.*

